# Age-different extent of resection for clinical IA non-small cell lung cancer: analysis of nodal metastasis

**DOI:** 10.1038/s41598-020-66509-5

**Published:** 2020-06-12

**Authors:** Han-Yu Deng, Jie Zhou, Ru-Lan Wang, Rui Jiang, Xiao-Ming Qiu, Da-Xing Zhu, Xiao-Jun Tang, Qinghua Zhou

**Affiliations:** Lung cancer center, West China Hospital, Sichuan University, Chengdu, China

**Keywords:** Surgical oncology, Non-small-cell lung cancer

## Abstract

Whether age has any impact on the risk of lymph node (LN) metastasis in patients with early-stage non-small cell lung cancer (NSCLC) remains controversial. Therefore, we aimed to objectively compare the risk of LN metastasis between elderly and young patients so as to justify for age-different extent of surgical resection for treating these patients. We retrospectively collected clinical data of patients undergoing lobectomy or segmentectomy with systematic hilar and mediastinal LN dissection for clinical stage IA peripheral NSCLC from January 2015 to December 2018. Both multivariate logistic regression analysis and propensity score-matched (PSM) analysis were applied to compare the risk of LN metastasis between elderly (>65 years old) and young (≤65 years old) patients. We finally included a total of 590 patients for analysis (142 elderly patients and 448 young patients). In the analysis of unmatched cohorts, young patients tended to have higher rates of hilar/intrapulmonary LN (13.4% VS 9.2%) and mediastinal LN metastasis (10.5% VS 6.3%) than elderly patients. In the multivariate analysis, age was found to be an independent predictor of both hilar/intrapulmonary (Odds ratio(OR) = 2.065, 95%confidence interval(CI): 1.049–4.064, P = 0.036) and mediastinal (OR = 2.400, 95%CI: 1.083–5.316, P = 0.031) LN metastasis. Moreover, in the analysis of well-matched cohorts generated by PSM analysis, young patients had significantly higher rates of hilar/intrapulmonary (18.8% VS 9.4%, P = 0.039) and mediastinal LN metastasis (17.1% VS 6.0%, P = 0.008) than elderly patients. Therefore, age remains to be an independent predictor of LN metastasis in early-stage NSCLC and age-different extent of surgical resection may be justified for these patients.

## Introduction

Lung cancer has become the leading cause of cancer and cancer-related death worldwide^1^.There are two major types of lung cancer, namely small cell lung cancer(SCLC) and non-small cell lung cancer(NSCLC), of which NSCLC accounted for about 85%^2^. With the advancement of computed tomography (CT), more and more early-stage NSCLCs are being found^3,4^. For the management of early-stage NSCLC, anatomic lung resection with systematic lymph node (LN) dissection (SLND) or sampling is recommended as the preferred option in the National Comprehensive Cancer Network guideline^5^. However, the extent of lung resection and lymphadenectomy for treating early-stage NSCLC depends largely on the characteristics of tumors such as tumor size, status of LN metastasis, histology as well as ground glass consistency^6^ and the patient’s physiological conditions.

It is reported that the age at diagnosis for majority of early-stage NSCLC patients falls into the range from 65 to 79 years old^7^ and the number of elderly patients continues to increase with the aging population. Moreover, significant difference of baseline characteristics between elderly and young lung cancer patients was observed^8^. However, current guideline still recommends the same surgical strategies for elderly and young patients except for elder patients with age-related or other co-morbidities which may lead to high risk of perioperative morbidity and mortality, of whom less extensive resection is suggested^5^. Hence, we are wondering whether there is evidence justifying age-different extent of surgical resection between elderly and young patients with early-stage NSCLC. As we know, from surgical perspectives, the optimal extent of surgical resection for early-stage NSCLC depends largely on the risk of intrapulmonary and mediastinal LN metastasis^9^. Therefore, it is of great value to compare the risk of LN metastasis between elderly and young patients with early-stage NSCLC so as to provide evidence justifying for age-different extent of surgical resection for elderly and young NSCLC patients. Previous studies comparing the risk of LN metastasis between elderly and young patients have reported different results due to significant patient selection bias^10,11^. Therefore, in this study, we aimed to draw an objective conclusion regarding the different risk of LN metastasis (both intrapulmonary/hilar and mediastinal LNs) between elderly and young patients by conducting both multivariate logistic regression analysis and propensity score-matched (PSM) analysis, so as to provide evidence for justifying age-different extent of surgical resection for elderly and young patients with early-stage NSCLC.

## Methods and Materials

### Patients

This was a retrospective study analyzing clinical and pathological data of patients receiving lobectomy or segmentectomy with SLND in our center between January 2015 and December 2018. In our center, the standard procedures of SLND were performed in accordance with The European Society of Thoracic Surgeons guideline^12^. In our center, contrast-enhanced chest and upper abdominal CT, brain magnetic resonance imaging, bone scanning, and cardiopulmonary tests were routinely carried out for preoperative assessment of lung cancer patients^13^. Positive LN metastasis was defined as LN with a short-axis diameter >1 cm on transverse chest CT scan. Routinely, in our center, we would not perform invasive preoperative staging for resectable lung cancer patients. We set the following inclusion criteria for patient inclusion: 1) patients should undergo upfront lobectomy or segmentectomy with SLND without any preoperative therapy; 2) patients should be preoperatively evaluated with clinical stage IA disease without positive LNs on preoperative CT scan(T1N0M0, tumor size ≤3 cm); 3) patients should have peripherally located lung cancer(defined as tumors in the outer one third of the hemithorax)^14^; 4) patients should be pathologically diagnosed as primary NSCLC. The exclusion criteria were also as follow: 1) patients who received preoperative chemoradiotherapy; 2) patients who were confirmed with synchronous multiple primary NSCLC or SCLC or secondary lung cancer.; 3) patients who had centrally located NSCLC; 4) patients who were intraoperatively found to have pleural metastasis. Our work was approved by the Ethics Committee of West China Hospital, Sichuan University. Because this study was a retrospective study and patients were analyzed anonymously in the study, the Ethic Committee of our hospital (West China Hospital, Sichuan University) waived the need for consent. Moreover, this study was carried out in accordance with the Declaration of Helsinki.

In this study, we analyzed the data of age, gender, radiographic features, pathologic findings, and LN metastasis status. The risk of LN metastasis was analyzed by age and patients were subgrouped into elderly group (>65 years old) and young group (≤65 years old) according to their age at diagnosis. Radiographic features included tumor location, tumor consistency (solid/part-solid/ground glass), and tumor size. Pathologic findings consisted of tumor histology and differentiation as well as the status of visceral pleural invasion(VPI). Patients in the study were staged based on the eighth edition of TNM staging system for NSCLC and LNs were further subgouped into N1(hilar/intrapulmonary LNs) and N2 LN stations(mediastinal LNs)^15^.

### Statistical analysis

All statistical analyses were conducted by using the IBM SPSS software (version 22.0; IBM Corp., Armonk, NY, USA). We presented continuous data as mean ± standard deviation and categorical data as number with percentage. Chi-squared test or Fisher’s exact test was used to compare categorical data between groups while independent-sample Student’s t-test or the Mann–Whitney non-parametric U-test was applied for comparing continuous data. We applied multivariate logistic regression analysis to explore potential predictors of LN metastasis (both intrapulmonary/hilar and medisatinal LNs) for these patients, which was based on our discretion by adding all clinically relevant variables. Moreover, we further performed PSM analysis using the PSMATCHING 3.04 and R-2.15.1-win software as previously described^16^ to further validate our results based on well-matched cohorts. The following covariates were used for matching: gender, tumor size, histology, tumor differentiation and consistency. Moreover, we used the nearest neighbor method to match elderly and young patients at a ratio of 1:1 with a caliper width of 0.01. Here, the statistical significance was set as a two-sided P-value of <0.05.

## Results

### Baseline characteristics of the patients included in the study

In total, 590 patients met our inclusion criteria and were enrolled in the study. The baseline characteristics of these patients are listed in Table 1. The mean age was 58.1 ± 10.9 years old and 56.6% of the patients were female. The mean tumor size was 1.90 ± 0.67 cm and majority of the tumors were pure solid nodules (66.3%) and located in bilateral upper lobes (63.7%). Most of the tumors were pathologically diagnosed as adenocarcinoma (89.5%) with the majority of them being lepidic (32.4%) and acinar (32.7%) predominant invasive adenocarcinoma and about half of the tumors were moderately differentiated (49.5%). Fifteen patients were confirmed as carcinoma *in situ*, 42 patients as minimally invasive adenocarcinoma, 56 patients as T1a disease, 163 patients as T1b disease, 152 patients as T1c disease, and 162 patients were upstaged as T2a disease due to VPI (27.5%). The mean number of total dissected LN was 12.3 ± 5.8 and it was 4.1 ± 3.2 for N1 LNs and 8.2 ± 4.1 for N2 LNs. As for LN metastasis, 14.1% of the patients were confirmed to have positive LNs with a N1 LN metastasis rate of 12.4% and N2 LN metastasis rate of 9.5%.

**Table 1:** Baseline characteristics of these clinical T1N0M0 peripheral non-small cell lung cancer patients stratified by age.

Characteristics	Total (N = 590)	Unmatched cohort			Matched cohort		
		Young group(≤65 years old, N = 448)	Elderly group (>65 years old, N = 142)	P value	Young group(≤65 years old, N = 117)	Elderly group (>65 years old, N = 117)	P value
Age (Mean ± SD,years)	58.1 ± 10.9	54.1 ± 9.0	71.1 ± 4.6	<0.001	54.6 ± 8.3	71.1 ± 4.6	<0.001
Gender				0.005			0.695
Male	256(43.3%)	180(40.2%)	76(41.7%)		56(47.9%)	59(50.4%)	
Female	334(56.6%)	268(59.8%)	66(46.5%)		61(52.1%)	58(49.6%)	
Tumor consistency				0.009			0.617
Pure ground glass	85(14.4%)	75(16.7%)	10(7.0%)		10(8.5%)	10(8.5%)	
Part-solid	114(19.3%)	80(17.9%)	34(23.9%)		21(17.9%)	27(23.1%)	
Pure solid	391(66.3%)	293(65.40%)	98(69.0%)		86(73.5%)	80(68.4%)	
Histology				0.008			0.729
Adenocarcinoma	528(89.5%)	409(91.3%)	119(83.8%)		98(83.8%)	98(83.8%)	
Lepidic group*	191(32.4%)	150(33.5%)	41(28.9%)		31(26.5%)	35(29.9%)	
Acinar predominant IA	193(32.7%)	147(32.8%)	46(32.4%)		37(31.6%)	35(29.9%)	
Papillary predominant IA	22(3.7%)	15(3.3%)	7(4.9%)		4(3.4%)	6(5.1%)	
Micropapillary predominant IA	87(14.7%)	73(16.3%)	14(9.9%)		21(17.9%)	12(10.3%)	
Solid predominant IA	35(5.9%)	24(5.4%)	11(7.7%)		5(4.3%)	10(8.5%)	
Squamous cell carcinoma	47(8.0%)	27(6.0%)	20(14.1%)		14(12.0%)	16(13.7%)	
Others	15(2.5%)	12(2.7%)	3(2.1%)		5(4.3%)	3(2.6%)	
Tumor location				0.305			0.985
Right upper lobe	220(37.3%)	162(36.2%)	58(40.8%)		45(38.5%)	46(39.3%)	
Right middle lobe	48(8.1%)	37(8.3%)	11(7.7%)		10(8.5%)	8(6.8%)	
Right lower lobe	84(14.2%)	66(14.7%)	18(12.7%)		16(13.7%)	15(12.8%)	
Left upper lobe	156(26.4%)	126(28.1%)	30(21.1%)		27(23.1%)	27(23.1%)	
Left lower lobe	82(13.9%)	57(12.7%)	25(17.6%)		19(16.2%)	21(17.9)	
Tumor size (Mean ± SD, cm)	1.90 ± 0.67	1.85 ± 0.69	2.04 ± 0.59	0.002	2.02 ± 0.64	2.00 ± 0.59	0.842
Tumor differentiation				0.031			0.614
G1	131(22.2%)	100(22.3%)	31(21.8%)		21(17.9%)	27(23.1%)	
G2	292(49.5%)	233(52.0%)	59(41.5%)		51(43.6%)	49(41.9%)	
G3	167(28.3%)	115(25.7%)	52(36.6%)		45(38.5%)	41(38.5%)	
pT stage				0.010			0.244
Tis	15(2.5%)	14(3.1%)	1(0.7%)		5(4.3%)	1(0.9%)	
T1mi	42(7.1%)	37(8.3%)	5(3.5%)		6(5.1%)	4(3.4%)	
T1a	56(9.5%)	50(11.2%)	6(4.2%)		7(6.0%)	6(5.1%)	
T1b	163(27.6%)	115(25.7%)	48(33.8%)		27(23.1%)	41(35.0%)	
T1c	152(25.8%)	110(24.6%)	42(29.6%)		33(28.2%)	31(26.5%)	
T2a	162(27.5%)	122(27.2%)	40(28.2%)		39(33.3%)	34(29.1%)	
pN stage				0.285			0.025
N0	507(85.9%)	382(85.3%)	125(88.0%)		90(76.9%)	104(88.9%)	
N1	27(4.6%)	19(4.2%)	8(5.6%)		7(6.0%)	6(5.1%)	
N2	56(9.5%)	47(10.5%)	9(6.3%)		20(17.1%)	7(6.0%)	
Visceral pleural invasion				0.827			0.480
Yes	162(27.5%)	122(27.2%)	40(28.2%)		39(33.3%)	34(29.1%)	
No	428(72.5%)	326(72.8%)	102(71.8%)		78(66.7%)	83(70.9%)	
Total dissected LN number (Mean ± SD)	12.3 ± 5.8	12.1 ± 5.7	12.9 ± 5.9	0.146	12.7 ± 5.7	12.5 ± 5.8	0.776
Total dissected intrapulmonary/hilar LN number (Mean ± SD)	4.1 ± 3.2	4.1 ± 3.1	4.0 ± 3.5	0.721	4.5 ± 3.3	3.9 ± 3.2	0.119
Total dissected mediastinal LN number (Mean ± SD)	8.2 ± 4.1	8.0 ± 4.1	8.9 ± 4.3	0.022	8.2 ± 3.9	8.6 ± 4.3	0.399
LN metastasis rate	83(14.1%)	66(14.7%)	17(12.0%)	0.410	27(23.1%)	13(11.5%)	0.015
N1 LN metastasis rate	72(12.4%)	60(13.4%)	13(9.2%)	0.181	22(18.8%)	11(9.4%)	0.039
N2 LN metastasis rate	56(9.5%)	47(10.5%)	9(6.3%)	0.141	20(17.1%)	7(6.0%)	0.008

Note: SD = standard deviation; invasive adenocarcinoma. *Lepidic group contains adenocarcinoma *in situ*, minimally invasive adenocarcinoma and lepidic predominant invasive adenocarcinoma.

### Comparison of baseline characteristics and LN metastasis rate between elderly and young patients

**Figure 1:**
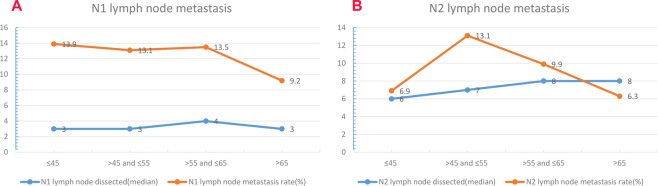
The median number of dissected lymph node and lymph node metastasis rate subgrouped by age: (**A**) N1 lymph node and (**B**) N2 lymph node.

The lymph node metastasis status of different age groups was presented in Fig. 1. All patients were further subgrouped into elderly group (>65 years old) and young group(≤65 years old) by age and there were 142 elderly patients and 448 young patients. The baseline characteristics and LN metastasis rate of the two groups were listed and compared in Table 1. The mean age for elderly and young patients were 71.1 ± 4.6 and 54.1 ± 9.0, respectively (P < 0.001). There were significantly more female patients (P = 0.005) and more ground-glass opacity (P = 0.009) as well as more adenocarcinoma (P = 0.008) in the young group than in the elderly group. Moreover, the mean size of the tumor in the elderly group was significantly larger than in the young group (P = 0.002) and significantly more poorly differentiated tumors were observed in the elderly group (P = 0.031). Elderly patients had significantly higher T stages than young patients (P = 0.010). However, there was no significant difference of tumor lobe location and VPI status between the two groups. Moreover, there was also no significant difference of number of total dissected LNs. As for LN metastasis, there was no significantly different LN metastasis rate between the two groups but young patients tended to have a higher rate of N1 (13.4% VS 9.2%, P = 0.181) and N2 (10.5% VS 6.3%, P = 0.141) LN metastasis than elderly patients.

A multivariate regression analysis was conducted to identify potential risk factor of LN metastasis (both for N1 and N2 LNs) in clinical IA NSCLC patients by entering relevant factors into multivariate analyzing model (Table 2). For the risk of LN metastasis, tumor consistency (P = 0.002), tumor size (P = 0.047), and tumor differentiation (P < 0.001) were found to be independent predictors. However, for the risk of N1 LN metastasis, age (OR = 2.065, 95%CI: 1.049–4.064, P = 0.036), tumor consistency (P = 0.001), histology (P = 0.033), and tumor differentiation (P < 0.001) were found to be independent predictors. For the risk of N2 LN metastasis, age (OR = 2.400, 95%CI: 1.083–5.316, P = 0.031), tumor consistency (P = 0.044), and tumor differentiation (P < 0.001) were also found to be independent predictors.

**Table 2:** Multivariate regression analysis for risk factors of lymph node metastasis among clinical stage IA (cT1N0M0) peripheral non-small cell lung cancer patients.

Characteristics	Odds Ratio	95% Confidence Interval	P value
LN metastasis			
Age (young VS elderly)	1.691	0.910–3.142	0.097
Gender (male VS female)	0.730	0.423–1.259	0.257
Tumor consistency (pure solid VS part-solid/pure ground glass)	4.505	1.713–11.850	0.002
Histology (adenocarcinoma VS nonadenocarcinoma)	0.764	0.372–1.564	0.463
Tumor size (cm)	1.603	1.007–2.551	0.047
Tumor differentiation (G3 VS G1/G2)	3.860	2.243–6.643	<0.001
Visceral pleural invasion (yes VS no)	1.529	0.899–2.601	0.117
N1 LN metastasis			
Age (young VS elderly)	2.065	1.049–4.064	0.036
Gender (male VS female)	0.770	0.435–1.362	0.369
Tumor consistency (pure solid VS part-solid/pure ground glass)	11.274	2.644–48.069	0.001
Histology (adenocarcinoma VS nonadenocarcinoma)	0.460	0.226–0.938	0.033
Tumor size (cm)	1.283	0.790–2.083	0.313
Tumor differentiation (G3 VS G1/G2)	2.779	1.587–4.867	<0.001
Visceral pleural invasion (yes VS no)	1.615	0.923–2.825	0.093
N2 LN metastasis			
Age (young VS elderly)	2.400	1.083–5.316	0.031
Gender (male VS female)	0.612	0.320–1.170	0.137
Tumor consistency (pure solid VS part-solid/pure ground glass)	3.108	1.033–9.349	0.044
Histology (adenocarcinoma VS nonadenocarcinoma)	1.422	0.555–3.640	0.463
Tumor size (cm)	1.516	0.867–2.652	0.145
Tumor differentiation (G3 VS G1/G2)	6.250	3.172–12.314	<0.001
Visceral pleural invasion (yes VS no)	1.738	0.935–3.232	0.080

Note: LN = lymph node.

### Comparison of LN metastasis rate between elderly and young patients in the matched cohorts

In order to actually compare the risk of LN metastasis between elderly and young patients, we conducted a PSM analysis to generate well-matched pairs by matching following unbalanced covariates: gender, tumor size, histology, tumor differentiation, and tumor consistency. Finally, we obtained a total of 117 pairs of well-matched patients for analysis. The baseline characteristics and LN metastasis rate of the two groups were listed and compared in Table 1. There were no significant difference of gender, tumor consistency, histology, tumor lobe location, tumor size, tumor differentiation, VPI status, and number of total dissected LNs (both N1 and N2 LNs) between the two well-matched groups. However, young patients were found to have a significantly higher rate of LN metastasis than elderly patients (23.1% VS 11.5%, P = 0.015). Moreover, there was significantly higher rate of N1 (18.8% VS 9.4%, P = 0.015) and N2 (17.1% VS 6.0%, P = 0.015) LN metastasis in the young patients than in the elderly patients.

### Comment

The status of LN metastasis remains to be an important factor determining the extent of lung resection and lymphadenectomy in the surgical management of early-stage NSCLC. However, the impact of age on the risk of LN metastasis remains controversial among previous literatures^10,11,17^. Therefore, we conducted this retrospective study aiming to figure out the actual impact of age on LN metastasis of early-stage NSCLC so as to provide objective evidence justifying for age-different extent of surgical resection for them. In this study, we compared the rate of LN metastasis between elderly (>65 years old) and young (≤65 years old) patients by including a total of 590 patients with clinical IA peripheral NSCLC. In the analysis of unmatched cohorts, we found that young patients tended to have a higher rate of N1 (P = 0.181) and N2 (P = 0.141) LN metastasis than elderly patients. However, significant patient bias such as gender, tumor size, histology, tumor differentiation, and tumor consistency was observed. Therefore, during multivariate regression analysis adjusting for above potential factors, age was found to be an independent predictor of N1 (OR = 2.065, 95%CI: 1.049–4.064, P = 0.036) and N2 (OR = 2.400, 95%CI: 1.083–5.316, P = 0.031) LN metastasis in patients with clinical IA peripheral NSCLC. Moreover, in the analysis of well-matched cohorts by PSM analysis, we further confirmed that young patients had a significantly higher risk of LN metastasis (both N1 and N2 LNs) than elderly patients.

Our findings agreed with the majority of previous studies concluding that young age was an independent predictor of LN metastasis in NSCLC^17–21^. However, some of the previous studies also showed that age did not significantly influence the risk of LN metastasis in NSCLC^11,22,23^. Possible reason for the discrepancy were the patient inclusion criteria, analytical methods, and sample size. For example, some studies only focused on clinical stage I NSCLC^11,22,23^ while others focused on resectable NSCLC (clinical stage I-III)^20^. Moreover, some studies calculated age as categorical data^17–21^ while others analyzed age as continuous data^10^. Nearly all these previous studies finding no correlation between age and LN metastasis had a relatively limited sample size (<300 cases)^11,22,23^. Therefore, it is reasonable that previous studies have drawn different conclusions about the comparison of LN metastasis between elderly and young patients.

Since there is no study directly comparing the risk of LN metastasis between elderly and young NSCLC patients available yet, we conducted such study for the first time by applying both multivariate regression analysis and PSM analysis. Both multivariate regression analysis and PSM analysis confirmed that young age was significantly correlated with LN metastasis (both N1 and N2 LNs), which provided objective evidence that young age was an independent predictor of LN metastasis in clinical IA NSCLC. Previously, young age was also confirmed to be an independent risk factor of brain metastasis in NSCLC patients^24^ and young patients were found to have a significantly higher rate of hematogenous dissemination of lung cancer cells during surgery^25^ and worse outcome of distant metastasis dynamics^26^ than elderly patients, indicating that lung cancer in young patients may exhibited more malignant and higher aggressive features than that in elderly patients. Moreover, previous experimental studies also found that age-related alterations of the extracellular matrix may lead to the less invasive behavior of lung cancer in aged animal models^27^. As a result, considering the significant different risk of LN metastasis and metastatic behavior between elderly and young patient, age-different extent of surgical resection may be indicated for early-stage NSCLC patients. Previous evidence from retrospective studies showed that in elderly patients with early-stage NSCLC, sublobar resection may yield equivalent long-term outcomes to lobectomy^28^. Even though more elderly patients received sublobar resection, they may yield similar cancer-specific survival to young counterparts^29^. Moreover, in young patients with early-stage NSCLC, lobectomy was found to confer better survival than sublobar resection^30^. Taken together, we think that it may seem justifiable to provide compromised limited lung resection and lymphadenectomy to elderly patients with early-stage NSCLC especially for those with chronologic comorbidities whereas for young counterparts standard lung resection and lymphadenectomy should always be anticipated. However, one should be cautious when deciding limited lung resection and lymphadenectomy for elderly patients since they still have the chance of nodal upstaging despite of a very low rate.

Our study had several limitations. First, our study was a retrospective analysis and the retrospective study design could limit our analytical power. Second, the conclusions were drawn only based on LN metastasis rate not on survival analysis. Moreover, positron emission tomography (PET)/CT was not commonly applied in our study and it is unclear how our data and results would apply to cohorts staged with PET/CT. Finally, the exact extent of limited lung resection and lymphadenectomy for elderly patients needs to be further established. Therefore, further prospective well-designed studies with survival analysis are warranted to confirm our conclusions.

## Conclusions

In this study, we objectively compared the risk of LN metastasis (both N1 and N2 LNs) between elderly and young patients with clinical stage IA peripheral NSCLC. Young patients were found to have a significantly higher risk of LN metastasis than elderly counterparts in both multivariate regression analysis and PSM analysis after adjusting for confounding factors. Therefore, young age was an independent predictor of LN metastasis in early-stage NSCLC and age-different extent of surgical resection may be indicated for treating these patients.
